# Muscle strength, physical fitness and well-being in children and adolescents with juvenile idiopathic arthritis and the effect of an exercise programme: a randomized controlled trial

**DOI:** 10.1186/1546-0096-11-7

**Published:** 2013-02-22

**Authors:** Eva Sandstedt, Anders Fasth, Meta Nyström Eek, Eva Beckung

**Affiliations:** 1 Department of Paediatrics, Institute of Clinical Sciences, University of Gothenburg, Gothenburg, Sweden; 2 Department of Neuroscience and Physiotherapy, Sahlgrenska Academy, University of Gothenburg, Gothenburg, Sweden

**Keywords:** Muscle strength, Grip strength, Physical fitness, Well-being, JIA, Exercise programme

## Abstract

**Background:**

Decreased muscle strength, fitness and well-being are common in children and adolescents with juvenile idiopathic arthritis (JIA) compared to healthy peers. Biological drugs have improved health in children with JIA, but despite this pain is still a major symptom and bone health is reported as decreased in the group. The improvement made by the biological drugs makes it possible to more demanding exercises. To jump is an exercise that can improve bone heath, fitness and muscle strength. The aim of the study was to see if an exercise programme with jumps had an effect on muscle strength, physical fitness and well-being and how it was tolerated.

**Methods:**

Muscle strength and well-being were studied before and after a 12-week exercise programme in 54 children and adolescents with JIA, 9–21 years old. The participants were randomized into an exercise and a control group. Muscle strength, fitness and well-being were documented before and after the training period and at follow-up after 6 months. Physical activity in leisure time was documented in diaries. The fitness/exercise programme was performed at home three times a week and included rope skipping and muscle strength training exercises.

Assessment included measurement of muscle strength with a handheld device, and with Grip-it, step-test for fitness with documentation of heart rate and pain perception and two questionnaires (CHAQ, CHQ) on well-being.

**Results:**

There were no differences between exercise and control group regarding muscle strength, grip strength, fitness or well-being at base line. Muscle weakness was present in hip extensors, hip abductors and handgrip. For the exercise group muscle strength in hip and knee extensors increased after the 12-week exercise programme and was maintained in knee extensors at follow-up. There was no change in fitness tested with the individually adapted step-test. The CHQ questionnaire showed that pain was common in the exercise group and in the control group. There were only small changes in the CHAQ and CHQ after the training period. The fitness/exercise programme was well tolerated and pain did not increase during the study.

**Conclusions:**

A weight bearing exercise programme, with muscle strength training with free weights and rope skipping was well tolerated without negative consequences on pain. It also improved muscle strength in the legs and can be recommended for children and adolescents with JIA.

## Background

Children and adolescents with juvenile idiopathic arthritis (JIA) in most parts of the world have decreased muscle strength, bone health and well-being compared to healthy peers [1-8]. The disease can affect school performance, physical training, family life, and activities in leisure time with peers [9-11]. Kimura et al. declare in a study from 2008 that pain is one of the major symptoms and limits the activities, disrupts school attendance and contributes to psychosocial distress [12].

The last decade has seen the introduction of biological drugs, e.g. anti-tumor necrosis factor alpha (anti-TNFα) also for paediatric rheumatic disorders [13,14]. The medical effect of anti-TNF is especially high in children with polyarticular onset of JIA [13,14]. In subjects with JIA the effects are described as improvement in functional ability, health-related quality of life, pain, sleep quality and daily participation and in terms of less flares or inflammatory active joints [15-17]. Anti-TNFα drugs are effective, safe and well tolerated in children with JIA [14-19].

Despite the use of biological agents, pain is reported as the major symptom of the disease, and joint pain is the leading cause of disability in this disease [12]. The authors describe pain perception as multifactorial and therefore require “a bio-psychosocial model that includes the individual’s age, developmental status, coping ability, mood, stress levels, and environmental and family factors, in addition to disease status and severity” [12].

Physical activity is important from a health perspective, especially in the subgroups with the polyarticular and extended oligoarticular categories [1,2,12,17,20]. Different physical activities have been studied such as jumping with a rope (rope-skipping) and exercise programs in water [4,6,7,21-24]. Jumping has influence on bone health and foot orthotics can significantly improve pain, speed of ambulation, and self-rated activity and functional ability [23,25].

Exercise programs with weight bearing exercises have been shown to improve both muscle strength and bone mass [6,22,23]. The exercise programmes in these studies were at different intensity levels and of different duration and physical activity in leisure time was not fully documented [4,6,22]. Takken showed that also cardiovascular fitness was decreased in children with JIA compared to healthy peers and point out the importance of cardiovascular fitness and motor performance as a part of total well-being [24]. Muscle strength is an important part in a fitness programme; muscle weakness in children with JIA is reported in many studies since the 1990s [2,8,9]. There is, however, a lack of knowledge about physical exercise levels and the impact on pain and well-being.

Physical fitness is described as a state of well-being with energy to participate in a variety of physical activities [26]. Frankala-Pinkham et al. stress the importance to incorporate more strategies to increase fitness, physical activity, and participation in the rehabilitation programme to improve quality of life (QoL) [27]. Questions concerning well-being and the impact of social and psychological functioning are well covered in the Child Health Questionnaire (CHQ) [28-30]. A couple of studies have reported that children with JIA after exercise interventions have less physical impairment or discomfort but report low levels of psychosocial abilities such as self esteem, psychosocial functioning and high levels of pain [21,22,25]. This seems to be a pattern for children with different chronic diseases or disabilities [31,32].

At our hospital the children with JIA attend the hospital regularly for physical training, which is time-consuming and costly both for the families and the health care system. An easy-to-handle home-based exercise programme making the patient less dependent on the physical therapist was needed, which was the impetus for this study. The aim of the study was to evaluate muscle strength, grip strength, physical fitness and well-being in a cohort of children and adolescents with JIA and the effects of a home-based exercise programme.

## Methods

### Subjects

**Figure 1:**
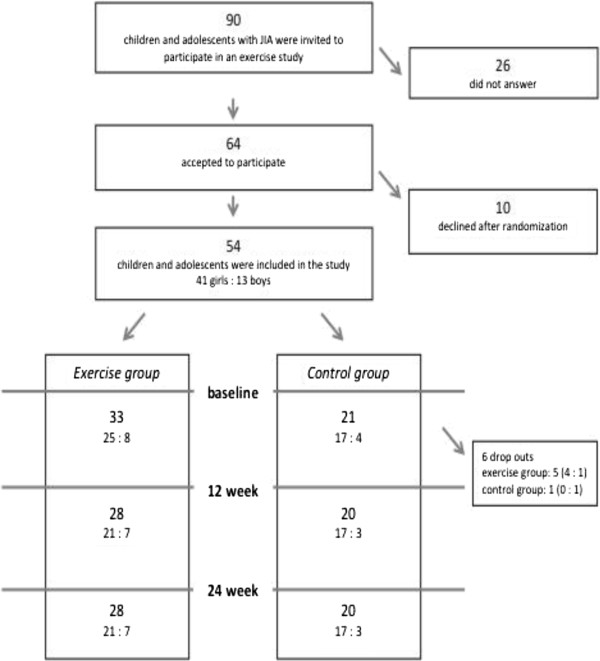
Flow chart of the randomization process and the test occassions.

The study is the second part of a randomized controlled trial of 54 children and adolescents with JIA that studied the effects of an exercise programme on bone health, muscle strength, fitness and well-being. Bone health and leisure time activities have been reported earlier and the randomization process has been described in detail [23]. The inclusion criteria were polyarticular or extended oligoarticular arthritis, treated with methotrexate, TNF-blockers and/or prednisone, and in need of repeated corticosteroid injections of joints in the lower extremities. Medical records were obtained and three participants were found to be diagnosed with enthesitis related and psoriatic arthritis. After written consent by the parents and assent by the children, the subjects were randomized into an exercise or a control group. The person carrying out the group allocation was blinded. A flow chart of randomization and training is presented in Figure 1. There were 10 dropouts from the control group after the randomization, as they had preferred to belong to the exercise group. There were another six dropouts after the first test occasion due to the families’ lack of time.

Muscle strength, range of motion, balance, fitness and well-being were studied before and after a 12-week exercise programme. The participants were evaluated three times; at base line, after 3 months at the end of the training period and at follow-up at 6 months. The same physiotherapist, who was blinded to the previous measurement, performed all measurements.

### Range of motion and balance

Range of motion (ROM) was measured with a plastic goniometer in dorsal and plantar flexion of the ankle; flexion, abduction and rotation of the hip; and rotation, abduction and flexion of the shoulder.

The Balance Reach Test for children [33] was performed before and after the exercise programme. It has a good test-retest and inter-rater reliability with intraclass correlation coefficients between 0.54 and 0.88 and 0.54 -0.93 respectively [33].

### Muscle strength

Muscle strength in arms and legs was tested with a handheld device (adapted Chatillon®, dynamometer; Axel Ericson Medical AB, Gothenburg Sweden), in eight muscle groups (shoulder abduction, elbow: extension, flexion, hip: extension, flexion, abduction, knee: extension, ankle: dorsal flexors) using the “make” technique and with standardized positions. After instruction and familiarisation with the procedure, three attempts were made and the maximum recording was used for data analysis. Lever arm for each muscle group was measured with a tape measure and torque was calculated (Nm). For six muscle groups the positions used in this study were similar to the one in a normative study [34], presenting equations for a predicted value for every muscle group based on age, sex and body weight. Muscle strength was compared with the normative material and calculated as a percentage of the predicted value. Thus it was possible to evaluate the whole group despite their different ages.

Grip strength was measured with Grippit (Detektor AB, Goteborg, Sweden) [35]. The instrument estimates peak strength over a 10 s period and the test was performed three times for each hand. The maximum recording was used for statistical analysis. Measurements were compared to normative values obtained with the same device [35], with data presented as mean ±1 SD and grouped according to age and sex. The data in this study was classified in a three level ordinal scale: *strong* = outside +1SD from mean, *average* = within mean ±1 SD and *weak* = outside −1 SD from mean.

### Physical fitness

Fitness was tested with a step-test. In the Harward step test from 1956 the step board was 45 cm high and the speed 30 steps per minute for 5 minutes or until exhaustion [36]. In this study the test was adapted, by using a lower step board (20 cm high), in order not to provoke pain. The participants were stepping on and off the step board for six minutes and a metronome was used for keeping an individually chosen speed. Heart rate was documented once a minute during the test and exertion was documented with the Borg Scale 6–20 [37]. The power in Watt was calculated taking into account body weight, gravity, the height of the step board and speed (*P = m × g × v*) and was normalised to body weight (W/Kg). The test was considered as an individual sub maximal test.

### Quality of life

The Child Health Assessment Questionnaire (CHAQ) is a questionnaire that is diagnose-specific for JIA, and is translated into and validated in Swedish [29]. The instrument refers to the last 14 days and includes eight different categories of activities (dressing, eating, walking, getting up, reaching, gripping, hygiene and activity). Each question is scored from 0 to 3 (0= no difficulty, 1= some difficulty, 2= much difficulty, 3= unable to complete task). The total score varies from 0 (no limitation) to 3 (extensive limitation). The instrument is recommended for children with JIA by the International League of Associations for Rheumatology (ILAR) and the Paediatric Rheumatology International Trials Organization (PRINTO) [29].

The Child Health Questionnaire (CHQ-C87) is a survey of the physical and psychosocial health of children 5 years of age and older. The questionnaire refers to the well-being status for the last four weeks. It was developed for children in the general population (for which normative data are available), and for children with chronic conditions [30]. The instrument has been validated for Swedish children, 9–16 years old, with epilepsy, diabetes and JIA [28]. It has a multidimensional profile consisting of 87 questions in twelve different domains (see Table 1). Scoring algorithms are provided for the different domains [30]. The manual consists of a Scale Scoring for a clinical sample of children with JIA, epilepsy, asthma and with psychiatric disorders.

**Table 1:** CHAQ and CHQ, mean and median at baseline

				**Exercise group n=32**		**Control group n=21**	
				**Mean**	**Median**	**Mean**	**Median**
***CHAQ***				.55	.50	.50	.38
***CHQ****domain*	*Nr of items*	*Low Score*	*High Score*				
Physical functioning	9	Child is limited a lot in performing all physical activities, including self-care, due to health.	Child performs all types of physical activities, including the most vigorous, without limitations, due to health	79.4	83.5	81.0	80.0
Role Emotional	3	Child is limited a lot in school work or activities with friends as a result of emotional problems	Child has no limitations in school work or activities with friends as a result of emotional problems	89.0	100.0	83.3	100.0
Role Behavioural	3	Child is limited a lot in school work or activities with friends as a result of behavioural problems	Child has no limitations in school work or activities with friends as a result of behavioural problems	89.9	100.0	94.6	100.0

### Pain

The children were asked to report if pain occurred during the test occasions. Presence of pain was documented with a 10 centimetres visual analogue scale (VAS). Pain in a perspective of health/well-being was also reported within the questionnaires CHAQ and CHQ.

### Fitness programme

The participants fulfilled a training programme three times a week for 12 weeks. The exercise programme consisted of rope skipping, muscle strength, core exercises and exercises with free weights for arms (Appendix). The programme has been described in detail earlier [23]. The number of repetitions performed was documented in an exercise diary. Physical activity in leisure time outside the programme was also documented in an activity diary.

### Statistical methods

For comparison between groups, Mann Whitney *U*-test was used for grip strength and for the questionnaires. *T*-test was used for muscle strength with myometer. The repeated measures ANOVA method was used for comparison of muscle strength and for step-test at baseline, after training and at follow-up. Data was tested with Mauchlys test of sphericity, and if sphericity was not assumed the Greenhouse-Geisser method/procedure was used for analysis.

As the results from the questionnaires were not normally distributed the Friedman test was used for repeated measures followed by post hoc testing with Wilcoxon singed rank test.

P-values of 0.05 or less were considered evidence of statistically significant findings. In the post hoc analysis Bonferroni adjustment for multiple comparisons was used.

Software packages Statview, SPSS (version 17.0) and SPSS for Mac (version 19.0) were used for statistical analysis.

### Ethics

This study was carried out in compliance with the Helsinki Declaration and was approved by The Regional Ethics Committee in Gothenburg. Written consent was obtained from the participants and from their parents.

## Results

Fifty-four children and adolescents were included in the study, with a mean age of 13.9 years (range 8.8-21.6). There were 41 girls and 13 boys, randomized in an exercise and in a control group (Table 2). There was a difference in age between the exercise group and the control group, not reaching statistical significance (p=0.059), but there were a statistically significant difference in height (p=0.007) and weight (p=0.026).

**Table 2:** Distribution of gender, age, height, weight, disease onset and type

	**Exercise group n=33**	**Control group n=21**
Girls *(*n=)	25 (76%)	17 (81%)
Age (years)	13.3 (8.8-19.9)	14.9 (8.8-20.6)
Height (cm)	153.4 (128.6-175.8)	163.8 (129–185.8) *
Weight (kg*)*	*47.9 (22.3-78)*	56 (35.8-82.9) *
Disease onset (years)	6.1 (1.2-16.5)	4.8 (1–13.4)
*JIA categories at onset*		
Polyarticular	20	9
Oligoarticular	7	8
Entesites related or psoriatic	1	3

* = statistically significant difference p<0.05.

### Range of motion and balance

There were no differences between groups at baseline for measurement of ROM in the Balance Reach Test and there were no significant changes during the study.

### Muscle strength

**Figure 2:**
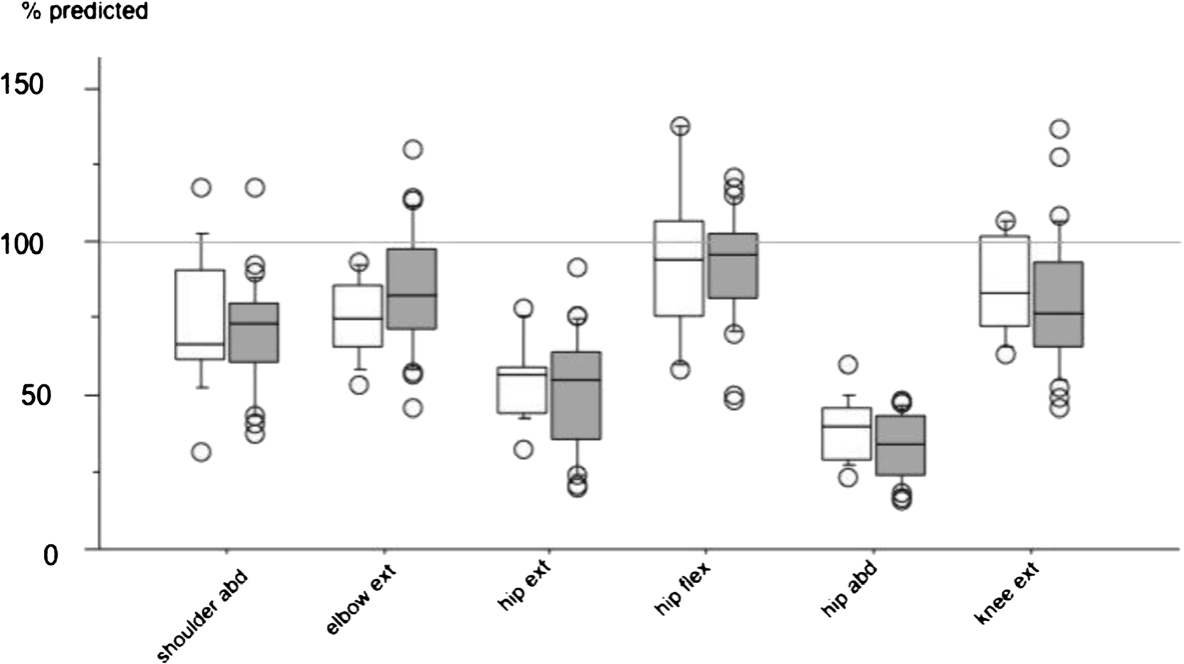
**Box plots showing muscle strength in percent of predicted value for six muscle groups in control group (white boxes) and exercise group (filled boxes (gray)).** Boxes indicate 25–75 percentiles where the horizontal line is the median, error bars indicate 10 or 90 percentiles and circles indicate outliers.

All children did not fulfil the whole protocol at all test occasions and only muscle groups with complete measurements were analysed. Muscle strength measurements taken at baseline were compared with the normative material for six muscle groups (see Table 3 and Figure 2). Values for hip abductors (33-38%) and hip extensors (52-55%) were below the limits of the 95% prediction interval. There were no significant differences between control and exercise group (Table 3). Values were also compared in order to see if age had influence on muscle strength. No significant differences were found when younger children (8–12 years) were compared with older children (13–16 years) (Figure 2).

**Table 3:** Muscle strength, expressed as a percentage of predicted value, mean (SD)

**Muscle –group**	**n**	**Exercise**	**n**	**Control**	***p-*value**^**a**^
Shoulder abductors	28	70.5 (17.6)	15	73.4 (21.6)	0.636
Elbow extensors	28	84.1 (19.6)	15	75.7 (12.7)	0.144
Hip extensors	27	52.1 (18.9)	15	55.9 (13.1)	0.496
Hip flexors	28	91.8 (17.8)	15	92.9 (25.5)	0.870
Hip abductors	28	33.9 (10.2)	15	38.8 (10.4)	0.138
Knee extensors	28	80.9 (21.5)	15	84.4 (15.4)	0.575

^a^ unpaired *t*-test.

Forty-five children had measurement of grip strength that could be compared to normative values [33], 17 in control and 28 in exercise group. The comparison showed weakness, with 28 children showing values below the normative mean −1 SD. Sixteen children were average (within 1 SD from mean) and one was strong with values above mean +1SD. There were no significant differences between groups when corrected for/compared to age (Table 4).

**Table 4:** Grip and muscle strength before, after training and at follow-up

		**Exercise group n=26**			**Control group n=19**		
		**Before**	**3 months**	**6 months**	**Before**	**3 months**	**6 months**
Grip strength	Right	157.9 (73.0)	173.4 (76.1)	174.4 (68.5)	197.1 (66.1)	208.8 (75.1)	216.9 (72.5)
	Left	150.5 (74.1)	149.1 (62.4)	155.1 (62.5)	185.4 (60.2)	185.1 (71.8)	194.0 (72.8)
*Muscle group*							
Shoulder abd	Right	21.8 (10.3)	23.3 (10.9)	24.0 (11.8)	26.5 (10.9)	25.5 (8.8)	30.2 (8.6) †
	Left	21.9 (10.2)	24.0 (11.3) *	23.9 (11.1)	25.0 (8.7)	25.5 (7.7)	27.0 (7.7)
Elbow ext	Right	21.6 (12.7)	21.9 (9.3)	23.4 (9.8)	26.0 (11.5)	25.4 (10.6)	26.7 (9.7)
	Left	21.1 (9.0)	22.2 (8.8)	23.0 (8.0)	24.9 (8.0)	25.8 (8.5)	26.3 (8.8)
Elbow flex	Right	22.2 (12.9)	24.8 (10.8)	26.5 (12.6) *	31.0 (14.1)	31.8 (12.0)	33.7 (13.2)
	Left	21.1 (11.6)	23.5 (10.9)	24.6 (10.9) *	30.0 (10.9)	31.5 (12.2)	31.1 (10.8)
Hip ext	Right	47.5 (21.4)	54.3 (22.4) *	49.1 (19.6)	66.7 (24.4)	64.0 (27.3)	60.7 (25.1)
	Left	47.3 (19.3)	56.1 (22.5) *	53.3 (20.4)	64.1 (20.1)	64.9 (25.0)	61.7 (22.9)
Hip flex	Right	53.5 (20.7)	56.0 (21.7)	54.1 (21.9)	68.8 (25.5)	64.3 (22.4)	66.2 (21.6)
	Left	49.9 (17.9)	53.9 (19.4)	52.4 (20.1)	62.0 (21.4)	60.8 (17.0)	63.4 (18.9)
Hip abd	Right	26.5 (13.6)	28.9 (15.1)	29.6 (10.6)	40.6 (18.3)	40.2 (15.5)	33.3 (9.7)
	Left	24.6 (12.3)	30.7 (15.3) *	32.9 (14.1) *	35.9 (12.3)	37.0 (14.1)	38.0 (12.8)
Knee ext	Right	57.3 (21.5)	65.8 (22.3) *	67.5 (22.3) *	79.3 (19.2)	78.7 (21.9)	77.1 (20.9)
	Left	55.8 (22.8)	61.7 (18.0) *	65.9 (26.4) *	78.8 (18.3)	79.6 (20.3)	78.8 (18.1)
Dorsiflex	Right	19.3 (5.2)	20.1 (6.3)	21.8 (6.1) *	23.8 (6.8)	22.1 (6.5)	25.2 (5.6) †
	Left	20.3 (5.5)	20.7 (6.7)	21.8 (5.8)	23.9 (6.5)	21.9 (4.8)	23.8 (5.0)

* = statistically significant difference to measurement 1 (baseline) p<0.03.

† = statistically significant difference to measurement 2 (after 12 week training period) p<0.03.

Data in Nm, mean (SD).

### Changes after fitness programme

There were no changes in grip strength during or after the training period (see Table 4).

Measurements of muscle strength of the legs are presented in Table 4. Statistically significant changes were found in the exercise group after training, with an increase in hip extensors and knee extensors, compared to baseline. Knee extensor strength was maintained at follow-up.

### Physical fitness

There were nine dropouts in the step-test due to pain; five experienced pain in the knee, one in the hip and three in the foot. The power in W/Kg and heart rate in the step-test are shown in Table 5. There were no differences between groups before training started regarding power and heart rate. There were no changes in heart rate or perceived exertion after training (Table 6).

**Table 5:** Results from the step-test: power and heart rate

	**Exercise group n = 33**	**Control group n = 19**	***p-*value**^**a**^
W/Kg	1.20 (1.43)	1.23 (0.17)	0.508
Heart rate	170.9 (16.14)	176.8 (19.8)	0.246

Comparison between groups before training, mean (SD).

**Table 6:** Step-test; heart rate and exertion at baseline and after 3 and 6 months, mean (SD)

	**Exercise group n=25**			**Control group n=17**		
	**Before**	**3 months**	**6 months months**	**Before**	**3 months**	**6 months months**
Heart rate	168.4 (16.4)	169.1 (15.2)	168.0 (16.9)	177.6 (20.9)	173.6 (23.6)	177.0 (18.4)
Borg scale	14.6 (2.86)	14.0 (3.03)	14.2 (3.58)	15.5 (1.97)	15.0 (3.00)	14.9 (2.89)

### QoL and well-being

Results of CHAQ and CHQ are shown in Tables 1 and 7. Fifty-three children completed the CHAQ and CHQ at baseline. There were no differences between the exercise and the control group. The CHQ was only used in scientific studies such as the study of Norrby [38]. The CHQ was used for all the participants in this study. The adolescents even the one 20.6 years of age and those older than 16 years were considered adolescents as the participants still were patients at the Children´s hospital. Our subjects showed low values in the domain “bodily pain” and also in the domains “general health” and “mental health” at baseline. 35 children fulfilled the CHAQ and 39 the CHQ at all test occasions. There was no increase in pain during the study. There were only small changes in both of the questionnaires. In the control group there was a statistically significant increase in CHQ domain “role physical” at the end of the study period. There was a tendency to improved “mental health” in the exercise group and deterioration in the control group in “general health”.

**Table 7:** CHAQ and CHQ

	**Exercise group**				**Control group**			
		**Baseline**	**3 months**	**6 months**		**Baseline**	**3 months**	**6 months**
		**Mean**	**Mean**	**Mean**		**Mean**	**Mean**	**Mean**
CHAQ	*n=20*	0.50	0.41	0.50	*n=15*	0.44	0.46	0.37
*CHQ domain*	*n=23*				*n=16*			
Physical functioning		83.4	88.4	87.6		80.4	86.9	84.63
Role Emotional		91.7	91.9	87.2		81.6	86.8	90.5
Role Behavioural		88.9	93.7	90.3		92.9	97.8	97.1
Role physical		91.2	94.2	89.5		80.2	89.8	96.3*
Bodily pain	*n=22*	59.3	71.8	64.1	*n=15*	56.7	56.7	64.7
Behaviour	*n=18*	82.7	82.5	83.6	*n=15*	84.5	82.4	86.5
Mental health		71.0	74.3	77.2		70.1	70.3	71.6
Self esteem		78.7	82.0	80.0		80.3	78.6	81.9
General health	*n=22*	67.2	70.6	70.6	*n=14*	66.7	59.2	69.7

* difference compared to baseline, p<0.017. Bonferroni adjustment for multiple comparisons.

Outcome in mean at baseline, after 3 and 6 months.

### Exercise programme

**Figure 3:**
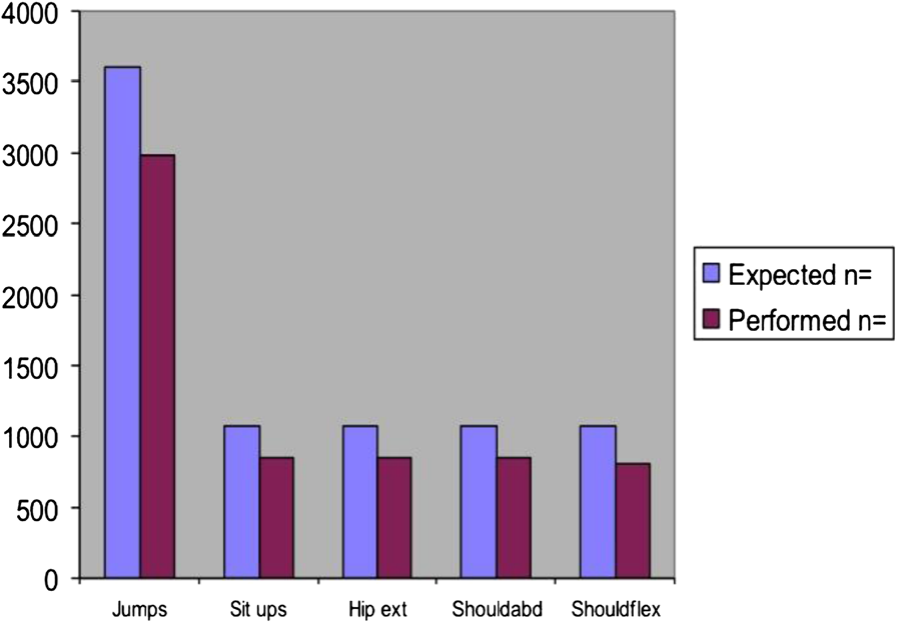
Numbers of expected and performed exercises in the exercise programme.

The participants in the exercise group fulfilled about 70% of expected numbers of exercises (Figure 3).

## Discussion

The study revealed muscle weakness and the presence of pain in children and adolescents with JIA. Pain is not an obstacle for performing the physical fitness programme, but there were ten dropouts mainly due to pain in the testing procedure.

### Muscle strength in the leg

Muscle weakness in knee extensors, elbow flexors and in ankle dorsal flexors has since 1995 been reported in children with JIA compared to healthy peers [2,3,7,8]. In our study muscle weakness was found in hip extensors, hip abductors and in handgrip strength. The other muscle groups were also below predicted values but within the normative range. In contrast to other studies our group was within normal values in knee extensors [3,7,8] Saarinen, Lindeman, and Broström all found that muscle weakness was present in children and adolescents with JIA, [3,7,8].

### Grip strength

More than 50% of the children in this study had a lower grip strength compared to peers and many of the children in the group had received corticosteroids in hands and fingers. No increase was found in hand grip strength after the training, which was not to be expected, as there were no specific exercises for grip strength in the programme. Earlier studies with children and adolescents report lower grip strength and that performance in writing and drawing in school give negative consequences compared to peers [39,40]. A pilot study shows that children with JIA are suffering from handwriting difficulties and are limited mainly due to pain and the inability to sustain handwriting for a longer period of time [40].

### Exercise programme

The increased muscle strength in hip and knee extensors found after 12 weeks exercise correlates well with the training programme that included of exercises for both hip and knee extensors. Rope skipping seemed to be an effective exercise to improve muscle strength in these muscle groups. Improvement in knee extensors is especially important, as the knee joint is the most affected joint in children and adolescents with JIA [10]. Earlier studies on groups of children and adolescents with JIA [1-4,20-25,38,41-43] show that this group is in inferior regarding functional ability, physical fitness and cardiovascular capacity compared to peers.

Earlier studies with rope skipping also reported significant improvement in bone health and muscle strength both for children with JIA and for healthy children [6,23,44]. The physical fitness programme also covered items for muscle strength in core muscles and muscles around the shoulders. The failure to increase muscle strength in these muscle groups may have been due to that the weights used were not heavy enough.

No changes in fitness, in terms of heart rate and exertion, were found after training. This may be due to the tests not being sensitive enough or the exercises were not sufficiently challenging regarding fitness. The step-test was performed on a 20 cm step board in order not to provoke knee pain; despite this ten children did not fulfil the test mainly due to pain in knee, hip or foot. A higher step board had been more demanding and could perhaps have shown a difference in the measurement. Measuring physical fitness with a cycle ergometer may be less painful for children with JIA. On the other hand the step-test with weight bearing exercise is a test close to functions that are important in daily life. Functional ability, physical fitness and cardiovascular capacity have earlier been studied in groups of children with JIA [20-24,40] who found that they were inferior regarding physical fitness compared to peers. There was a lack of knowledge regarding to the participants’ ability to perform the programme and how well they adhered to it. The focus had been on daily activity and of participating in physics at school. The focus had not been on progressive cardiovascular fitness.

With the improvement in medical treatment it is important to keep addressing physical fitness, as it is a prerequisite for good health [45,46].

The exercise programme in our study was designed to meet the need for physical training, in accordance to the ILAR recommendation. The frequency of three times a week and the level of cardiovascular effort and weights were well tolerated. In this study we did not individualize the programme or increase the number of repetitions or the load during the training period, which might have given another outcome.

Noteworthy, our protocol did not render any increase in pain during the training period. By the introduction of an exercise programme we also hoped to encourage the children to change from a sedentary to an active life style. As reported earlier, physical activity increased in the group during the study and at follow-up [23].

Out of the 54 participants 48 completed the training, but there were dropouts in some of the measurements. This was explained by; lack of time, pain, and different psychosocial reasons.

### Well-being

The questionnaires showed that the children in the study did not have any difficulties to carry out daily activities.

Compared to an earlier study in the same region and compared to the results from normative data [38] our group scored higher on the domain “general health” but were at about the same levels on the other domains. There were only a few changes during the 6-month period. The CHAQ has been debated because of lack of sensitivity in view of changes in rehabilitation [29]. Dempster et al. in a study from 2001 showed that a minimal clinically important improvement is represented by a median change of −0, 13 in the CHAQ [47]. They also found a minimal clinically important deterioration by a median change of 0.75. In our study no improvement or deterioration was found.

Our results confirm that it is not an optimal questionnaire for children with a moderate impairment and being in an inactive phase of the disease, as it did not capture the spontaneous positive comments from participants during the study.

In our study the children reported low levels in the domain “bodily pain” and also in the domains “general health” and “mental health”. The pathogenesis of pain in children with rheumatic diseases is multifactorial, and disease treatment alone is often not enough to alleviate it. Many researchers stress that children with chronic conditions can have “hidden” consequences on self esteem and well-being why pain treatment should include non-pharmacological interventions, for example exercise and cognitive-behavioural therapy, for better outcome on their general health [12,17,45,46].

## Conclusions

Muscle weakness was present in hip extensors, hip abductors and in the handgrip. Muscle strength in hip and knee extensors increased after the 12-week exercise programme and was maintained in knee extensors at follow-up. Pain was common in the group. The exercise programme was well tolerated, there was a compliance of 70% to the programme and pain did not increase during the study.

The study shows that a weight bearing fitness programme with muscle strength training including free weights and rope skipping can be recommended for children and adolescents with JIA.

## Abbreviations

anti-TNFα: anti Tumor Necrosis Factor alpha; CHAQ: Child Health Assessment Questionnaire; CHQ: Child Health Questionnaire; ILAR: International League of Association for Rheumatology; JIA: Juvenile Idiopathic Arthritis; Nm: Newton metre; PRINTO: Peadiatric Rheumatology International Trials Organization; QoL: Quality of Life; ROM: Range of movement; VAS: Visual Analogue Scale; W/kg: Weight/kilogram.

## Competing interests

The authors declare that they have no competing interests.

## Authors’ contributions

ES carried out the conception and design, all measurements (except DXA), performed analyses, supervised the training period and drafted the manuscript. AF participated in the design of the study and was involved in drafting the manuscript. MNE performed statistical analyses, provided reference data and was involved in drafting the manuscript. EB participated in the design of the study and drafted the manuscript. All authors have read and approved the final manuscript.
